# Exploring Aachen minipigs as in vivo model for human intracranial studies: Focus on hind limb artery diameters

**DOI:** 10.1371/journal.pone.0320606

**Published:** 2025-03-27

**Authors:** Lara Bender, Thorsten Sichtermann, Christoph Dorn, Jan Minkenberg, Andrea Stockero, Rebecca May, Hani Ridwan, Charlotte S. Weyland, Omid Nikoubashman, Martin Wiesmann, Christiane Franz

**Affiliations:** Department of Diagnostic and Interventional Neuroradiology, University Hospital RWTH Aachen, Aachen, Germany; Universidade de Trás-os-Montes e Alto Douro: Universidade de Tras-os-Montes e Alto Douro, PORTUGAL

## Abstract

Endovascular methods for stroke treatment have become increasingly popular in recent years and continue to be improved. Pigs serve as in vivo models in endovascular stroke research for device testing and training purposes. Due to a network of small vessels between the cerebral and intracranial arteries, called rete mirabile, catheterization of the brain-supplying arteries in pigs is impossible. Alternatively, various porcine arteries can be used. Aachen minipigs have become more popular in the last years, but specific data on their vascular diameters are limited to the forelimb arteries. Therefore, the aim of our study was to assess diameters of porcine hind limb arteries in eight female Aachen minipigs (weight of 47.08 kg ±  4.58 kg and age of 17 to 21 months), focusing on the internal iliac artery, external iliac artery, femoral artery, profunda femoris artery, popliteal artery, caudal tibial artery, cranial tibial artery, and sacral median artery, and to compare these diameters with diameters of human brain-supplying arteries. Measurements of artery diameters were conducted during experimental digital subtraction angiographies of a superordinate endovascular study. We found similarities between diameters of porcine hind limb arteries and human brain-supplying arteries: the porcine cranial tibial artery, sacral median artery, caudal tibial artery, popliteal artery, and profunda femoris artery are suitable for modelling human arteries. Our work may serve as a tool for planning and conducting interventional experiments involving Aachen minipigs and may therefore lead to reduced animal numbers according to the 3Rs (Replacement, Reducement, Refinement) from Russell and Burch.

## Introduction

Endovascular methods for stroke treatment have become increasingly popular in recent years and continue to be improved [1, 2]. Pigs serve as in vivo models in endovascular stroke research for the evaluation of medical devices and training of interventional radiologists [3–5]. The suitability of pigs for endovascular research arises from their anatomical and hemodynamic similarity to the human vascular system, as well as their comparable body size to humans [6–8].

For acute endovascular experiments and training purposes, German Landrace pigs with a weight of 45-60 kg at the age of five to six months are commonly used [3,9–12]. German Landrace pigs are less suitable for chronic experiments because they grow rapidly, which makes handling more difficult and the vessel diameters do not remain stable over the duration of chronic experiments [13, 14]. For chronic experiments, adult minipigs are more suitable, because of their stable body weight and vessel diameters [6,14–16].

A special characteristic of the vessel anatomy of the porcine brain is the rete mirabile, a network of small arteries between the cerebral and intracranial arteries [3,4,9]. Due to the rete mirabile, catheterization of the porcine brain-supplying arteries is impossible. Alternatively, various porcine arteries can be used in endovascular research, e.g., the subclavian or visceral arteries [8,12,15]. In order to increase the number of suitable vessels, the vessels of the porcine hind limbs are of potential interest. While several authors report diameters of the hind limb arteries of domestic swine and Göttingen minipigs, data about Aachen minipigs are lacking [8,14,17,18]. Siefert et al. investigated the diameters of the thoracic and abdominal aorta including hind limb arteries in juvenile Göttingen minipigs of 8-12 weeks, but not in adult Aachen minipigs [17]. To the best of our knowledge, there are no studies that compare the diameters of hind limb arteries in adult minipigs with those of human brain-supplying arteries. Therefore, the aim of this study was to measure the diameters of the hind limb arteries in Aachen minipigs and compare them with the diameters of human brain-supplying arteries.

## Materials and methods

### Experimental design and animals

In this retrospective study, eight female Aachen minipigs (Gerd Heinrichs, Heinsberg-Karken, Germany) with a weight of 47.08 kg ±  4.58 kg (mean, standard deviation) and an age of 17 to 21 months were included. All minipigs were part of another neuroendovascular study. Therefore, our work contributes to the principle of the 3Rs (Replacement, Reducement, Refinement) according to Russell and Burch [19].

The experiments complied with the German Animal Welfare Law and the EU Directive 2010/63/EU. The governmental animal care and use committee (Landesamt für Natur, Umwelt und Verbraucherschutz (LANUV) Nordrhein Westfalen, Recklinghausen, Germany) approved the experimental protocol, the corresponding approval number being AZ-81-02.04.2019.A412. The animals were housed in compliance with the requirements of Appendix III of EU Directive 2010/63/EU and the Appendix of the European Agreement of March 18, 1986. Institutional guidelines for animal welfare and experimental implementation were followed.

### Animal preparation and procedures

Animal housing, feeding, and anesthesia were performed as previously described. The animals were fasted one day prior to experimental angiographies but had access to water. Euthanasia at the end of the experiments was also conducted as previously described [20]. In brief, the procedures were as follows:

All procedures were carried out under general anesthesia, including two digital angiographies during the course of the superordinate study. As part of the standard anesthetic protocol, premedication was administered consisting of azaperone (Stresnil, 40 mg, ad us. vet.; Sanochemia Pharmazeutika AG, Neufeld, Austria), atropine sulfate (Atropinsulfat; B. Braun Melsungen AG, Melsungen, Germany), and ketamine (10% Ketavet, ad us. vet.; Zoetis Deutschland GmbH, Berlin, Germany). Subsequently, orotracheal intubation was performed, and a urinary bladder catheter was placed non-surgically. Mechanical ventilation was provided using a controlled air-oxygen mixture throughout the anesthesia period. Depending on the experimental requirements, anesthesia was sustained using either isoflurane (1.5 vol%) or propofol (Propofol 2% MCT Fresenius; Fresenius Kabi Deutschland GmbH, Bad Homburg, Germany). To ensure adequate analgesia, fentanyl (Fentanyl-Janssen, 0.5 mg; Janssen-Cilag GmbH, Neuss, Germany) was administered in all cases. The animals remained in a supine position throughout anesthesia, with body temperature continuously regulated within physiological limits. Physiological parameters, including heart rate, electrocardiogram (ECG), and oxygen saturation (monitored via pulse oximetry), were continuously assessed during anesthesia. Postoperative recovery was closely observed, and analgesic treatment with carprofen (Rimadyl, ad us. vet.; Zoetis Schweiz GmbH, Zürich, Switzerland) was provided for 24 hours. At the end of the experiments, euthanasia was carried out by intravenous injection of sodium pentobarbital (Narcoren 16g/100ml; Merial GmbH, Hallbergmoos, Germany) while the animals were under anesthesia.

### Image acquisition and vessel diameter measurement

**Fig 1:**
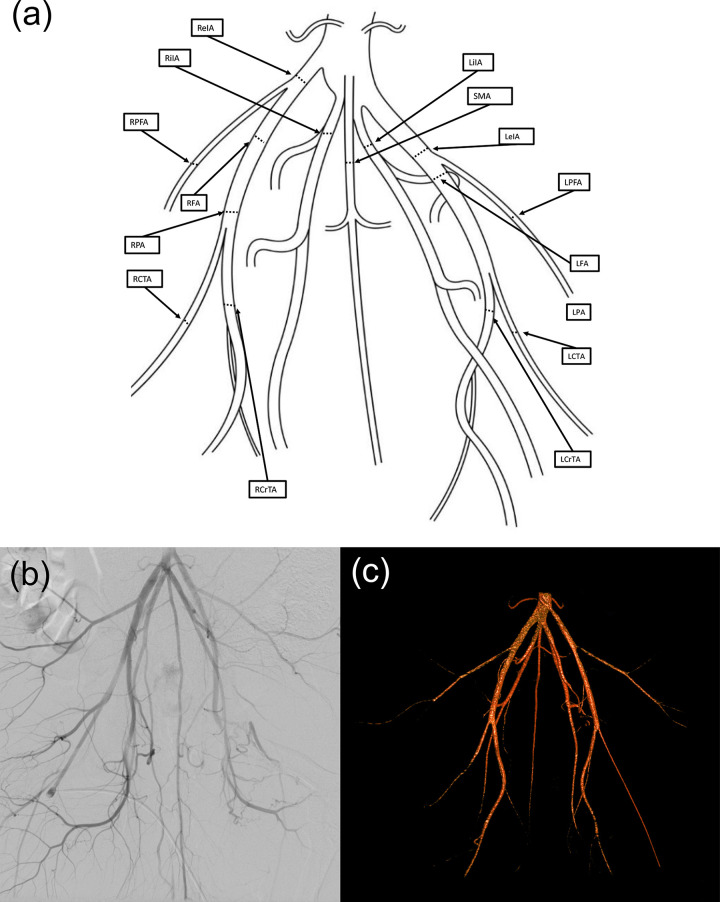
Anatomy of the porcine hind limb arteries. (a) Schematic drawing of the main branches of the porcine hind limb arteries. Diameters were measured at the black lines in the right and left internal iliac artery (RiIA, LiIA), right and left external iliac artery (ReIA, LeIA), right and left femoral artery (RFA, LFA), right and left profunda femoris artery (RPFA, LPFA), right and left popliteal artery (RPA, LPA), right and left caudal tibial artery (RCTA, LCTA), right and left cranial tibial artery (RCrTA, LCrTA), and sacral median artery (SMA). (b) Digital subtraction angiography of the left and right porcine hind limb arteries. (c) Angiography-based three-dimensional reconstruction of the left and right porcine hind limb arteries.

During experimental angiographies, a 6F sheath was inserted into the femoral artery of the anesthetized and analgesized minipig [20]. After flushing with heparinized saline, standard 5F or 6F catheters and guidewires were used to access various vessels, including the right and left subclavian arteries, common carotid artery, and the hind limb arteries. Digital subtraction angiography (DSA) was performed administering Iopamidol (Solutrast 300, 300 mg/ml; Bracco Imaging Deutschland GmbH, Konstanz, Germany) as contrast agent with a maximum dose of 5 ml per kg bodyweight. For image acquisition C-arm angiography units were used: Either a Siemens Arcadis Avantic/Arcadis ORBIC (Siemens Healthcare GmbH, Erlangen, Germany) or a Ziehm C-arm (Ziehm Vision, Ziehm Imaging GmbH, Nürnberg, Germany). The measurements of vessel diameters were conducted using the Picture Archiving and Communication System (PACS) (IntelliSpace PACS 4.4 Enterprise, Philips GmbH, Eindhoven, The Netherlands), focusing on the internal iliac artery (iIA), external iliac artery (eIA), femoral artery (FA), profunda femoris artery (PFA), popliteal artery (PA), caudal tibial artery (CTA), cranial tibial artery (CrTA), and sacral median artery (SMA) (Fig 1). We measured vessel diameters of each vessel segment at 1 cm after the respective vessel origin. The accuracy of measurements was ensured by a careful calibration process based on the size of the guide catheter.

For the comparison of porcine hind limb arteries and human brain-supplying arteries, vessel diameters of 100 acute stroke patients were used as reported previously [15]. In summary, data were obtained from CT angiography and DSA examinations. This cohort included 45 female and 55 male patients, ages ranging from 3 to 105 years (mean, 71 years). This retrospective data analysis was performed after evaluation by the local ethics board of our institution (Ethik-Kommission an der Medizinischen Fakultät der Rheinisch-Westfälischen Technischen Hochschule Aachen (RWTH Aachen)). Data were accessed between November 5, 2021 and November 19, 2021. The authors had no information that could identify individual participants during or after data collection. All methods were carried out in accordance with relevant guidelines and regulations.

As previously described, measurements were taken at predefined human vessel segments, including the internal carotid artery (ICA carotid-T), middle cerebral artery (MCA, M1 segment), M2 segments of the MCA (M2 superior trunk, M2 inferior trunk), anterior cerebral artery (ACA, A1 segment), vertebral artery (VA, intracranial and terminal segment of VA), and basilar artery (BA, proximal segment and below basilar top). The porcine vessel segments included the internal iliac artery (iIA), external iliac artery (eIA), femoral artery (FA), profunda femoris artery (PFA), popliteal artery (PA), caudal tibial artery (CTA), cranial tibial artery (CrTA), and sacral median artery (SMA).

### Statistical analysis

The mean, standard deviation, median, and interquartile range (IQR) were determined for each diameter. Data were tested for normal distribution using the Shapiro-Wilk test. Mann-Whitney U tests were performed to compare the diameters of the right and left hind limb arteries in minipigs and to compare the arterial diameters of pigs and humans. To address the multiple comparisons, we used the Bonferroni correction, considering a corrected p-value less than 0.001 as significant. Statistical analyses were conducted using SPSS Statistics 28 software (IBM, San Jose, California, USA).

## Results

**Fig 2:**
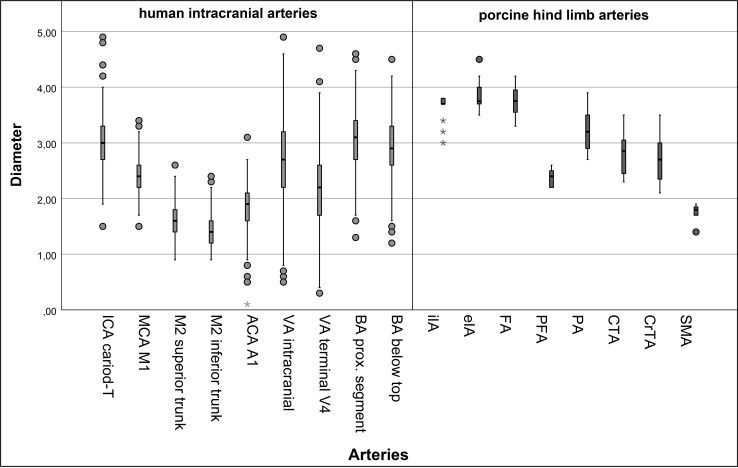
Box-and-whisker plot of human intracranial (bright grey) and porcine hind limb (dark grey) arterial diameters (in millimeters). Human vessels: internal carotid artery (ICA), middle cerebral artery, (MCA, M1), M2 segments of the middle cerebral artery (M2 superior trunk and inferior trunk), anterior cerebral artery (ACA), vertebral artery (VA), and basilar artery (BA). Porcine vessels: internal iliac artery (iIA), external iliac artery (eIA), femoral artery (FA), profunda femoris artery (PFA), popliteal artery (PA), caudal tibial artery (CTA), cranial tibial artery (CrTA), and sacral median artery (SMA).

In this study, the diameter of seven different paired arterial branches and one unpaired vessel (SMA) of the hind limb arteries were measured in 8 minipigs, resulting in 15 measurements per pig and a total of 120 measurements. In addition, we measured 9 segments of human brain-supplying vessels each within our cohort of 100 stroke patients. All values except those of the ICA, MCA (M2 segments), ACA, VA segments, eIA, FA, PA, CTA and CrTA were normally distributed. The measurements of porcine artery diameters are presented in Table 1 and Fig 2. Among the different porcine vessel sections, the largest vessel diameters were found in the eIA and the FA, each measuring 3.8 mm (mean). The smallest vessel diameter was found in the SMA at 1.8 mm. Among the different porcine vessel segments, no significant differences in vessel diameters were found between the left and right hind limb arteries (p > 0.382). In human brain-supplying arteries, the ICA had the largest diameter, measuring 3.0 mm (mean), while the M2 segment (inferior trunk) had the smallest diameter at 1.4 mm (Fig 2).

**Table 1:** Arterial diameters of human intracerebral arteries and porcine hind limb arteries in millimeters according to quantitative vascular angiography.

Human intracranial arteries	Mean ± 1 SD	Median (IQR)	Min - Max
ICA, carotid-T	3.0 (±0.5)	3.0 (2.7-3.3)	1.5-4.9
MCA, M1 segment	2.4 (±0.4)	2.4 (2.2-2.6)	1.5-3.4
MCA, M2 segment, superior trunk	1.6 (±0.3)	1.6 (1.4-1.8)	0.9-2.6
MCA, M2 segment, inferior trunk	1.4 (±0.3)	1.4 (1.2-1.6)	0.9-2.4
ACA, A1 segment	1.8 (±0.4)	1.9 (1.6-2.1)	0.1-3.1
VA, intracranial	2.7 (±0.9)	2.7 (2.2-3.2)	0.5-4.9
VA, terminal segment	2.1 (±0.8)	2.2 (1.7-2.6)	0.3-4.7
BA, proximal segment	3.0 (±0.7)	3.1 (2.7-3.4)	1.3-4.6
BA, below basilar top	2.9 (±0.6)	2.9 (2.6-3.3)	1.2-4.5
**Porcine hind limb arteries**	Mean ± 1 SD	Median (IQR)	Min-Max
iIA	3.7 (±0.2)	3.7 (3.7-3.8)	3.0-3.8
eIA	3.8 (±0.3)	3.8 (3.7-4.0)	3.5-4.5
FA	3.8 (±0.3)	3.8 (3.5-3.9)	3.3-4.2
PFA	2.4 (±0.1)	2.4 (2.2-2.5)	2.2-2.6
PA	3.2 (±0.4)	3.2 (2.9-3.5)	2.7-3.9
CTA	2.8 (±0.4)	2.9 (2.4-3.1)	2.3-3.5
CrTA	2.7 (±0.4)	2.7 (2.3-3.0)	2.1-3.5
SMA	1.8 (±0.3)	1.8 (1.7-1.9)	1.4-1.9

**Human vessels:** ICA: internal carotid artery, MCA: middle cerebral artery, ACA: anterior cerebral artery, VA: vertebral artery, and BA: basilar artery.

**Porcine vessels**: iIA: internal iliac artery, eIA: external iliac artery, FA: femoral artery, PFA: profunda femoris artery, PA: popliteal artery, CTA: caudal tibial artery, CrTA: cranial tibial artery, and SMA: sacral median artery.

### Comparisons of porcine and human artery diameters

The diameter of the human intracranial VA segment was comparable with four porcine hind limb arteries: PFA (p = 0.028), PA (p = 0.002), CTA (p = 0.626), and CrTA (p = 0.932). The diameter of the human terminal VA segment was comparable with the PFA (p = 0.115), the CrTA (p = 0.001), and the SMA (p = 0.029). The diameter of the human BA segment was comparable with three porcine segments: PA (p = 0.220), CTA (p = 0.060), and CrTA (p = 0.029). The diameter of the BA segment below the basilar top was also comparable with the PA (p = 0.021), CTA (p = 0.322), and CrTA (p = 0.184). The diameter of the human ICA segment was comparable with three porcine segments: PA (p = 0.048), CTA (p = 0.075), and CrTA (p = 0.028). The diameter of the MCA (M1 segment) was comparable to the PFA (p = 0.910) and the CrTA (p = 0.003). The diameters of the M2 superior (p = 0.060) and inferior trunk segments (p = 0.001), and ACA were comparable to the SMA (p = 0.250). These results are illustrated in Table 2.

**Table 2:** Presentation of the Mann-Whitney U-test results (p-values) for differences of artery diameters between porcine hind limb arteries and human intracerebral arteries.

Artery	iIA	eIA	FA	PFA	PA	CTA	CrTA	SMA
ICA, carotid T	<0.001	<0.001	<0.001	<0.001	**0.048**	**0.075**	**0.028**	<0.001
MCA, M1, segment	<0.001	<0.001	<0.001	**0.910**	<0.001	<0.001	**0.003**	<0.001
MCA, M2, superior trunk	<0.001	<0.001	<0.001	<0.001	<0.001	<0.001	<0.001	**0.060**
MCA, M2, inferior trunk	<0.001	<0.001	<0.001	<0.001	<0.001	<0.001	<0.001	**0.001**
ACA, A1 segment	<0.001	<0.001	<0.001	<0.001	<0.001	<0.001	<0.001	**0.250**
VA, intracranial	<0.001	<0.001	<0.001	**0.028**	**0.002**	**0.626**	**0.932**	<0.001
VA, terminal segment	<0.001	<0.001	<0.001	**0.115**	<0.001	<0.001	**0.001**	**0.029**
BA, proximal segment	<0.001	<0.001	<0.001	<0.001	**0.220**	**0.060**	**0.029**	<0.001
BA, below basilar top	<0.001	<0.001	<0.001	<0.001	**0.021**	**0.322**	**0.184**	<0.001

Non-significant differences between artery diameters are marked in bold.

## Discussion

When working with in vivo models, detailed knowledge on the specific model and careful selection of the animal model are necessary to achieve the study objectives. By comparing the diameters of porcine hind limb arteries with human brain-supplying arteries, we found similarities between human and porcine arteries. We measured a relatively wide range of porcine artery diameters from 1.8 to 3.8 mm. This was in line with our expectations, as we used eight different arteries of the porcine hind limb vasculature. The human brain-supplying arteries ranged in diameter from 1.4 to 3.0 mm, which corresponds well to the porcine artery diameters. For this reason, we were able to select several porcine arteries that can serve as in vivo model for human brain-supplying arteries. For instance, the porcine CrTA, SMA, CTA, PA, and PFA are suitable for modelling human arteries.

Literature research revealed a study with similar results for the femoral artery (FA) diameter in Göttingen minipigs compared to our data: Hiebl et al. reported FA diameters of approximately 3.8 mm in Göttingen minipigs at the same age (17-21 month), which compares well to our results: We measured diameters of 3.8 ±  0.3 mm (mean, SD). Remarkably, these Göttingen minipigs had considerably lower body weights (approximately 26 to 34 kg) than the Aachen minipigs at the same age of our study with body weights of 47.1 kg ±  4.6 kg (mean, SD) [14]. However, the experimental setup included a different selection of vessels. Therefore, only the FA could be compared with our dataset.

Other arteries of our dataset, for example the iIA and eIA, were investigated in two studies with larger pig breeds with comparable body weight to the Aachen minipigs of our study [8,18]. Edwards et al. investigated the vascular anatomy of Yorkshire pigs of 40 to 60 kg and yielded diameters of the iIA and the eIA ranging from 4.4 to 5.6 mm and 6.0 to 6.8 mm, respectively [8]. Both the iIA and eIA provided higher diameters in the Yorkshire pigs than we measured in Aachen minipigs, resulting in ranges of 3.0 to 3.8 mm for the iIa and 3.5 to 4.5 mm for the eIa. In another study, Goes et al. report diameters of the eIA with values of 6.7 and 7.2 mm (mean, right and left side, respectively), and diameters of the iIA with values of 4.1 and 4.3 mm in three landrace pigs. The measurements of our study yielded mean values of 3.7 and 3.8 mm (iIA and eIA, respectively), which means that the diameters of the iIA are slightly higher and diameters of the eIA are considerably higher in landrace pigs compared to Aachen minipigs. The fact that the landrace pigs used in the study of Goes et al. and the Aachen minipigs of our study had comparable body weights, underlines the variability in artery diameters among the different pig breeds. This is particularly true with regards to the rapid growth of landrace pigs [15]. Therefore, the results of larger and fast-growing pig breeds cannot simply be extrapolated to pigs of other breeds with the same body weight.

To the best of our knowledge, no data on the other arteries we measured in Aachen minipigs, e.g., PFA, PA, CTA, CrTA, and SMA have been published. Therefore, a comparison with other pig breeds for these vessels was not possible. As Aachen minipigs are gaining more popularity in biomedical research and the complexity of neuroendovascular procedures increases, there is a need of further characterization of Aachen minipigs as endovascular in vivo model. Our work may serve as a tool for planning and conducting interventional experiments involving Aachen minipigs and may therefore lead to reduced animal numbers in the future.

## Limitations

For this study, the suitability of the hind limb arteries was assessed on the basis of the artery diameter. However, possible influencing factors such as histologic characteristics or physiologic parameters, e.g., blood pressure or blood flow patterns were not considered and could be a target for future studies. In addition, tortuosity of target vessel segments or arterial access vessels has an impact on the relevance of an experimental interventional model. The neurointerventionalists involved in our study had no objections to the suitability of the measured vessels in this regard. However, tortuosity was not analyzed as an additional parameter. Furthermore, we did not investigate the clinical tolerability of using the porcine hind limb vessels in endovascular experiments, because this was not the subject of the superordinate study. Therefore, no conclusion can be drawn about the use of porcine hind limb vessels in chronic trials. Investigations on specific endovascular settings with respect to animal welfare aspects would be desirable.

## Conclusion

In conclusion, we described the porcine hind limb artery diameters of the Aachen minipig, and we were able to identify several arterial branches that are suitable as in vivo model for human brain-supplying arteries.

## Supporting information

S1 Table Porcine artery diameters.
(PDF)

S2 Table Human artery diameters.
(PDF)
