# Supplementary material for: Exploring Aachen minipigs as in vivo model for human intracranial studies: Focus on hind limb artery diameters
Source: PLoS One. 2025 Mar 27;20(3):e0320606. doi: 10.1371/journal.pone.0320606 (PMC11949345; doi:10.1371/journal.pone.0320606)
Supplement: S1 Table — (PDF) [file pone.0320606.s001.pdf]

Case	Vessel left	Vessel right	Vessel left	Vessel right
	internal iliac artery	internal iliac artery	external iliac artery	external iliac artery
1	3.2	3.0	4.2	4.5
2	3.4	3.7	3.9	3.5
3	3.8	3.7	4.0	4.1
4	3.8	3.8	4.0	3.8
5	3.8	3.7	3.7	3.7
6	3.8	3.7	3.7	3.9
7	3.8	3.8	3.6	3.7
8	3.7	3.7	3.7	3.5
**Case**	**Vessel left**	**Vessel right**	**Vessel left**	**Vessel right**
	femoral artery	femoral artery	profunda femoris artery	profunda femoris artery
1	4.0	4.0	2.5	2.6
2	4.0	3.5	2.5	2.5
3	3.9	4.2	2.2	2.2
4	3.7	3.7	2.2	2.4
5	3.6	3.9	2.2	2.3
6	3.8	3.4	2.2	2.3
7	3.6	3.3	2.4	2.4
8	3.5	3.9	2.5	2.6
**Case**	**Vessel left**	**Vessel right**	**Vessel left**	**Vessel right**
	popliteal artery	popliteal artery	caudal tibial artery	caudal tibial artery
1	3.7	3.5	3.5	3.3
2	3.0	2.8	2.4	2.3
3	2.9	2.9	2.9	2.5
4	2.7	3.2	2.6	2.4
5	2.9	3.6	2.5	2.4
6	3.5	3.1	2.9	2.9
7	3.4	3.9	2.8	3.0
8	3.2	3.3	3.2	3.1
**Case**	**Vessel left**	**Vessel right**	**unpaired**	
	cranial tibial artery	cranial tibial artery	medial sacral artery	
1	3.1	3.0	1.9	
2	2.2	2.1	1.8	
3	2.3	2.9	1.8	
4	2.5	2.1	1.4	
5	2.4	2.7	1.7	
6	3.0	2.9	1.8	
7	3.5	3.5	1.9	
8	2.7	2.7	1.7	
