# Supplementary material for: Exploring Aachen minipigs as in vivo model for human intracranial studies: Focus on hind limb artery diameters
Source: PLoS One. 2025 Mar 27;20(3):e0320606. doi: 10.1371/journal.pone.0320606 (PMC11949345; doi:10.1371/journal.pone.0320606)
Supplement: S2 Table — (PDF) [file pone.0320606.s002.pdf]

Case	Vessel left	Vessel right	Vessel left	Vessel right
	ICA, carotid-T	ICA, carotid-T	MCA M1	MCA M1
1	3.4	3.2	2.9	3.2
2	3.3	2.1	3.1	3.4
3	2.9	3.1	2.5	2.3
4	3.5	3.2	2.5	2.2
5	3.2	3.6	3.1	2.5
6	4.4	3.8	2.9	2.2
7	2.7	2.2	2.5	2.5
8	2.5	2.9	2.1	2.6
9	3.6	2.6	2.9	3.3
10	2.7	3.1	2.4	1.8
11	3.1	3.2	1.9	2.9
12	2.2	3.1	2.1	2.6
13	2.4	2.7	2.0	2.8
14	3.8	2.4	1.9	1.9
15	4.0	2.6	2.3	2.6
16	3.2	2.7	2.4	2.2
17	3.0	2.6	2.5	2.6
18	3.6	3.4	2.3	2.2
19	4.2	3.2	2.2	2.3
20	3.1	2.8	2.8	2.5
21	3.5	2.0	2.3	2.1
22	2.5	2.7	2.7	2.3
23	3.1	2.1	2.7	2.1
24	3.2	2.9	2.3	2.0
25	3.0	3.9	2.8	3.1
26	2.6	2.7	2.3	2.4
27	3.4	2.8	2.4	2.6
28	3.2	2.9	2.8	2.1
29	3.5	1.5	2.2	2.5
30	3.6	3.6	2.5	2.2
31	3.7	3.2	2.4	2.7
32	2.5	3.1	2.2	2.5
33	2.5	3.3	2.4	2.4
34	2.7	3.3	2.1	2.6
35	1.9	2.7	1.8	1.5
36	3.1	4.4	2.6	2.5
37	3.1	2.9	2.3	2.2
38	2.7	2.7	2.2	2.3
39	2.8	2.1	2.0	2.0
40	3.5	2.0	2.9	2.3
41	2.4	2.2	2.2	1.7
42	2.4	2.4	1.9	2.1
43	2.8	2.8	2.4	1.9
44	2.9	3.8	1.8	2.4
45	3.3	3.6	2.7	2.6
46	3.6	3.1	2.3	2.2
47	3.9	2.8	2.3	2.1
48	2.9	2.7	2.5	2.5
49	2.7	3.3	2.2	2.2
50	2.8	2.3	1.8	1.9
51	4.9	3.1	3.4	2.6
52	3.5	3.2	2.9	1.8
53	2.6	2.8	2.1	1.9
54	4.2	2.5	1.8	2.5
55	2.9	2.3	2.5	2.6
56	2.6	2.9	2.2	2.2
57	2.6	3.2	2.3	2.2

58	2.4	3.2	2.4	2.8
59	3.1	2.8	2.8	2.2
60	3.4	2.5	2.4	1.8
61	2.8	2.7	2.4	2.4
62	4.0	2.5	2.6	1.9
63	2.9	2.5	1.9	1.8
64	3.9	3.6	2.6	2.4
65	2.9	3.2	2.1	2.3
66	3.6	2.6	2.3	2.1
67	2.7	2.4	2.2	2.2
68	3.9	3.5	2.4	2.2
69	2.6	3.0	2.2	2.5
70	2.7	3.0	2.2	2.5
71	3.0	3.2	2.4	2.3
72	4.8	3.0	2.6	2.9
73	2.9	3.0	1.9	2.2
74	3.3	3.4	2.2	2.1
75	2.8	2.9	2.1	2.3
76	3.4	3.2	2.5	2.2
77	2.5	3.1	2.3	2.3
78	3.2	3.4	2.5	2.3
79	3.0	2.9	2.4	2.4
80	2.4	2.3	2.1	1.9
81	2.3	2.2	1.7	1.5
82	3.2	3.2	1.9	2.6
83	3.5	3.5	2.8	2.8
84	2.9	2.9	2.4	2.4
85	3.0	2.9	1.7	1.9
86	2.9	3.0	2.0	2.0
87	3.4	3.4	2.6	2.5
88	3.1	3.0	2.5	2.6
89	3.1	3.0	2.7	2.6
90	3.5	3.3	2.9	2.7
91	3.0	2.9	2.5	2.4
92	3.1	3.0	2.8	2.7
93	2.9	2.9	2.7	2.5
94	2.4	2.4	2.0	1.7
95	3.4	3.6	2.9	2.5
96	2.6	2.7	2.5	2.3
97	3.1	3.0	2.9	2.4
98	2.9	2.8	2.8	2.9
99	3.3	3.1	2.6	2.7
100	2.8	2.9	2.5	2.6
	**Vessel left**	**Vessel right**	**Vessel left**	**Vessel right**
	M2 superior trunk	M2 superior trunk	M2 inferior trunk	M2 inferior trunk
1	1.7	1.7	1.2	1.5
2	1.5	1.6	1.3	2.0
3	1.2	1.3	1.1	1.8
4	1.2	1.8	1.7	1.4
5	2.0	1.6	1.8	1.3
6	2.1	1.4	1.6	1.2
7	1.6	1.4	1.7	1.8
8	1.1	1.8	1.4	1.1
9	2.4	2.6	2.1	2.1
10	1.4	1.2	1.6	1.4
11	1.9	1.7	1.3	1.9
12	1.3	1.3	1.6	1.5
13	1.8	1.5	1.1	1.7
14	1.3	1.2	1.9	1.7

15	1.6	1.6	1.3	1.8
16	1.2	1.9	1.5	1.6
17	1.6	1.2	1.4	1.6
18	1.6	2.3	1.6	1.3
19	2.1	1.8	2.2	2.4
20	1.7	1.8	2.3	1.1
21	1.9	1.9	1.5	1.4
22	2.4	1.6	1.6	1.9
23	2.1	1.5	1.3	1.8
24	1.3	1.8	1.8	1.3
25	1.9	2.3	1.1	1.6
26	1.7	1.3	1.5	1.1
27	1.6	1.9	1.5	1.2
28	1.9	1.7	1.1	1.3
29	1.2	1.1	1.8	2.0
30	1.9	1.4	1.4	1.2
31	1.8	1.2	1.3	1.5
32	1.4	1.4	1.0	1.6
33	1.2	1.6	1.5	1.1
34	1.4	1.9	1.1	1.6
35	2.1	1.2	2.1	1.4
36	2.1	1.6	2.2	2.1
37	1.4	1.6	1.5	1.6
38	1.6	1.5	1.5	1.6
39	1.4	1.2	1.6	1.4
40	1.5	1.7	1.3	1.1
41	1.2	1.6	1.1	1.2
42	1.4	1.8	1.2	1.3
43	1.4	1.6	1.6	1.4
44	1.3	1.3	1.2	1.5
45	1.7	1.8	2.1	1.4
46	1.4	1.5	1.7	1.4
47	1.9	1.9	1.3	2.0
48	1.9	1.4	1.2	1.8
49	1.7	1.8	1.1	0.9
50	1.4	1.6	1.1	1.4
51	2.1	2.0	1.7	1.4
52	1.7	1.6	1.2	1.1
53	1.3	1.7	1.4	1.2
54	1.2	1.9	1.9	1.3
55	2.1	1.2	1.3	1.7
56	1.4	1.4	1.2	1.6
57	1.1	1.7	1.3	1.2
58	1.3	1.8	1.9	1.2
59	1.8	1.6	1.4	1.5
60	1.9	1.4	1.3	1.2
61	1.8	1.6	1.4	1.4
62	1.8	1.6	1.2	1.4
63	1.7	1.2	1.2	1.6
64	1.8	1.8	1.4	1.6
65	1.7	1.7	1.2	1.4
66	2.1	1.8	1.6	1.2
67	1.7	1.7	1.4	1.4
68	1.5	1.4	1.7	1.6
69	2.0	1.7	1.4	1.6
70	1.8	1.6	1.4	1.1
71	1.7	1.8	1.1	1.3
72	1.9	1.7	1.4	1.4
73	1.6	0.9	1.4	1.3

74	1.8	1.8	1.2	1.2
75	1.7	1.6	1.3	1.5
76	1.3	1.6	1.1	1.5
77	1.8	1.6	1.4	1.1
78	1.8	1.7	1.3	1.4
79	1.4	1.2	1.1	1.8
80	1.5	1.6	1.2	1.0
81	1.2	1.1	0.9	0.9
82	1.3	1.2	1.1	1.4
83	1.8	2.0	1.1	1.2
84	1.7	1.8	1.2	1.2
85	1.3	1.3	1.1	1.1
86	1.4	1.3	1.1	1.0
87	1.9	1.8	1.2	1.2
88	1.8	1.4	1.3	1.2
89	1.6	1.5	1.3	1.7
90	1.4	1.7	1.2	1.3
91	1.1	1.7	1.4	1.1
92	1.9	1.2	1.2	1.8
93	1.1	1.2	1.5	1.4
94	1.1	1.4	1.3	1.1
95	1.2	1.4	1.4	1.1
96	1.2	1.3	1.1	1.5
97	1.8	1.2	1.1	1.8
98	1.7	1.7	1.1	1.2
99	1.5	1.6	1.1	1.3
100	1.7	1.6	0.9	1.1

	**Vessel left**	**Vessel right**	**Vessel left**	**Vessel right**
	ACA A1	ACA A1	VA intracranial	VA intracranial
1	1.6	1.6	3.3	4.6
2	2.1	1.7	3.2	2.8
3	1.9	1.6	3.4	2.4
4	2.3	2.6	2.8	2.2
5	2.2	2.3	2.3	2.4
6	2.1	2.4	3.0	2.3
7	1.9	1.8	2.0	1.9
8	1.8	1.4	2.7	2.2
9	2.4	2.7	3.1	3.5
10	1.8	2.0	2.3	2.5
11	2.1	1.9	3.8	2.6
12	1.9	2.1	2.2	2.2
13	2.0	2.3	2.6	3.4
14	2.1	2.3	3.3	2.0
15	2.2	2.2	3.1	4.0
16	2.2	2.2	2.2	2.9
17	2.4	2.5	2.7	2.5
18	2.1	1.4	3.3	2.1
19	1.7	1.2	3.9	2.5
20	2.1	1.3	3.5	2.4
21	2.2	1.8	2.2	2.8
22	1.9	1.7	3.8	2.4
23	2.4	2.0	3.4	2.6
24	1.9	2.1	2.0	3.5
25	0.8	2.7	2.5	2.6
26	2.1	1.8	1.2	1.7
27	2.1	2.1	4.2	3.5
28	2.0	2.1	2.1	2.8
29	1.2	1.8	2.6	2.6
30	1.9	2.2	2.4	3.1

31	1.8	2.4	2.1	2.4
32	1.4	2.1	3.3	3.5
33	2.4	2.4	3.1	4.0
34	2.1	2.5	3.1	2.5
35	0.9	1.9	1.8	2.2
36	2.3	2.3	3.1	1.2
37	3.1	2.2	2.1	2.5
38	n.a.	2.3	3.1	3.6
39	2.0	2.1	3.4	4.2
40	1.6	2.5	2.8	3.1
41	1.4	1.7	0.8	2.4
42	1.3	2.1	3.3	3.3
43	1.7	1.8	2.4	2.8
44	1.9	2.7	3.9	3.6
45	2.1	2.2	3.1	4.4
46	2.6	2.3	2.2	3.2
47	2.4	2.1	3.4	1.5
48	0.5	2.1	2.8	2.5
49	1.6	2.1	3.0	3.3
50	2.5	2.0	2.8	3.0
51	2.1	2.1	4.9	1.7
52	2.4	2.0	2.6	3.7
53	0.6	1.5	2.6	3.2
54	1.5	1.8	3.4	2.9
55	2.1	1.5	2.7	4.3
56	1.6	1.5	4.1	1.4
57	1.5	2.0	3.2	1.2
58	1.9	2.4	2.4	3.6
59	1.6	1.7	1.2	2.9
60	1.4	1.8	2.5	3.1
61	1.5	2.1	3.3	3.4
62	2.2	1.6	1.9	4.9
63	1.6	1.8	2.0	3.2
64	1.6	1.8	4.4	0.5
65	1.9	2.1	3.0	4.5
66	0.1	1.9	3.2	3.2
67	1.8	2.1	3.3	3.7
68	2.1	2.1	3.8	2.8
69	2.0	1.5	2.6	3.3
70	2.0	1.5	2.5	2.5
71	1.9	1.7	3.7	1.1
72	2.4	1.2	2.9	3.2
73	1.5	1.3	0.9	3.3
74	2.0	2.0	2.9	2.9
75	1.2	1.9	2.7	3.0
76	1.3	1.3	3.0	1.5
77	1.9	1.8	2.7	2.2
78	1.5	1.5	0.9	2.5
79	2.1	1.5	3.1	2.8
80	1.2	1.4	3.6	0.8
81	1.6	1.5	0.6	2.2
82	1.5	1.5	3.0	2.7
83	2.2	2.0	3.0	2.6
84	1.8	1.9	2.8	2.8
85	1.9	0.5	1.8	1.8
86	1.8	1.9	2.8	0.7
87	1.7	1.7	1.5	2.5
88	1.4	1.8	2.5	2.5
89	1.9	1.8	2.2	2.8

90	1.9	1.8	1.9	2.1
91	1.8	1.9	0.8	1.2
92	1.4	1.6	2.1	2.5
93	1.8	1.1	0.5	2.0
94	1.2	1.3	1.1	2.6
95	0.5	1.6	1.0	3.0
96	1.8	1.4	3.1	2.1
97	2.1	1.6	1.4	2.5
98	2.2	1.7	1.1	1.7
99	0.6	0.8	1.1	3.7
100	2.0	1.9	1.6	1.1
	**Vessel left**	**Vessel right**	**unpaired**	**unpaired**
	VA terminal V4	VA terminal V4	BA prox. Segment	BA below segment
1	3.2	4.1	4.6	4.1
2	3.0	2.3	3.1	2.9
3	2.7	1.6	2.2	2.4
4	2.5	1.6	2.8	2.7
5	2.2	1.9	3.4	2.9
6	2.9	2.8	3.9	3.7
7	1.5	1.6	3.1	2.7
8	2.0	1.8	2.9	3.5
9	2.2	2.9	3.5	2.8
10	2.4	2.0	2.9	2.9
11	3.5	2.2	4.5	4.5
12	1.1	2.3	2.1	2.1
13	1.8	2.1	2.7	1.9
14	1.8	1.4	2.0	2.5
15	1.5	2.2	4.3	2.7
16	1.8	2.7	2.4	2.8
17	2.4	2.2	3.3	3.3
18	2.5	3.8	2.7	2.2
19	2.8	2.4	3.9	3.8
20	2.4	2.2	2.7	2.7
21	0.8	3.5	3.5	3.1
22	3.3	2.3	2.9	3.4
23	2.3	1.8	2.6	2.6
24	1.8	2.7	3.7	3.4
25	2.3	2.2	2.9	2.9
26	0.9	1.3	2.2	2.1
27	2.9	2.2	4.6	4.2
28	2.0	2.6	3.6	3.4
29	2.3	1.8	3.4	3.4
30	1.8	2.2	2.7	2.7
31	1.7	1.5	2.8	2.3
32	2.1	2.5	3.4	3.0
33	1.6	2.4	2.4	2.4
34	2.8	2.2	3.1	3.1
35	0.5	2.2	1.9	1.5
36	2.8	0.9	3.6	2.6
37	1.9	2.1	2.8	2.6
38	1.4	2.7	2.2	1.8
39	2.8	2.1	3.8	2.4
40	2.5	1.6	3.2	3.5
41	1.4	2.5	1.7	1.6
42	2.7	2.1	2.6	2.7
43	2.4	2.4	2.8	2.8
44	3.3	2.9	2.9	2.4
45	1.9	3.6	3.4	3.4
46	2.6	3.1	3.6	3.7

47	2.8	1.5	2.8	2.7
48	2.1	2.2	3.4	3.1
49	2.1	2.7	3.8	3.6
50	2.8	2.0	3.0	2.9
51	4.7	0.8	3.9	3.6
52	2.1	2.9	3.7	3.8
53	2.7	2.0	3.2	3.2
54	2.3	3.0	3.2	3.0
55	2.6	2.2	3.3	3.4
56	3.9	1.4	3.9	3.6
57	2.0	0.5	2.9	2.9
58	2.4	1.9	3.0	3.0
59	0.6	2.1	2.6	2.5
60	2.3	1.8	3.2	3.0
61	2.4	2.3	3.2	3.2
62	1.5	3.8	3.3	3.3
63	1.6	2.5	2.8	2.6
64	3.6	0.5	4.6	3.0
65	1.5	3.6	3.9	3.7
66	2.6	1.8	3.6	3.6
67	2.6	2.7	3.3	3.0
68	2.6	2.9	2.9	2.8
69	2.5	3.1	2.8	2.9
70	2.2	2.2	3.3	3.1
71	3.4	0.7	3.4	3.3
72	1.9	2.8	3.6	4.0
73	0.5	2.7	1.9	2.0
74	2.3	2.0	3.1	3.0
75	2.2	3.0	3.2	3.1
76	2.8	0.9	3.3	3.2
77	2.5	1.8	2.8	2.7
78	0.7	2.2	1.8	2.0
79	2.5	2.3	3.1	3.0
80	3.0	0.6	2.4	2.4
81	0.3	1.9	1.9	1.7
82	2.8	2.5	3.3	3.3
83	2.8	2.3	3.5	3.5
84	2.1	2.4	3.4	3.3
85	1.5	1.0	1.6	1.4
86	2.0	0.4	3.1	2.8
87	0.8	2.2	2.7	2.5
88	2.4	2.2	3.2	3.1
89	1.8	2.5	3.1	3.0
90	1.6	1.9	3.3	3.0
91	0.5	1.2	1.8	1.8
92	1.7	1.9	2.9	2.8
93	0.5	1.7	1.3	1.2
94	0.6	2.1	2.5	2.5
95	0.7	2.8	2.9	3.0
96	2.9	1.9	3.0	2.9
97	0.9	2.2	3.1	3.0
98	0.7	1.6	2.4	2.3
99	1.0	2.7	2.8	2.7
100	1.6	1.3	3.0	2.9
